# ROS: their roles in diminished ovarian reserve

**DOI:** 10.3389/fendo.2026.1823452

**Published:** 2026-04-23

**Authors:** Xin Chen, Danzhuo Liu

**Affiliations:** Department of Obstetrics and Gynecology, School of Integrated Traditional Chinese and Western Medicine, Hunan University of Chinese Medicine, Changsha, China

**Keywords:** antioxidant therapy, dor, follicular development, os, ROS

## Abstract

Diminished Ovarian Reserve (DOR) is a core pathological condition leading to reduced female fertility, characterized by decreased follicular quantity and diminished oocyte quality in the ovaries, which significantly impacts natural pregnancy and assisted reproductive technology outcomes. Despite its complex etiology, recent studies have demonstrated that reactive oxygen species (ROS)-mediated oxidative stress (OS) serves as a common pathological hub, integrating multiple pathogenic factors, including aging, metabolic abnormalities, environmental exposure, and iatrogenic damage, throughout the process of DOR development. This article systematically reviews the key mechanisms of ROS in DOR and the corresponding intervention strategies. At the level of pathological mechanisms, ROS drive ovarian reserve depletion through a multilevel, networked mechanism. Based on mechanistic understanding, intervention strategies focus on mitochondrial-targeted antioxidants. This article provides a systematic framework for an in-depth understanding of the central role of ROS in DOR and the development of mechanism-driven intervention strategies.

## 1 Introduction

DOR refers to a decrease in the quantity and/or quality of oocytes remaining in the ovary. Clinically, DOR can be accompanied by symptoms such as frequent menstruation, scanty menstrual flow, amenorrhea, and even infertility. The World Health Organization (WHO) defines couple infertility as the failure to conceive after 12 consecutive months of regular unprotected sexual intercourse. WHO estimates the infertility rate from 1990 to 2021 at 17% (1). The infertility rate in China increased from 7.5% in 2007 to 18.2% in 2020 (2). Although the average life expectancy of modern societal populations continues to increase, the increase in infertility rates may be due to the fact that the ovaries age faster than other organs (3, 4). Therefore, the problem of ovarian disease is more serious than other organ diseases. The specific causes leading to DOR are not fully understood. Besides age being an independent risk factor for DOR, long-term sedentary behavior, lack of exercise, increased exposure to radiation, and environmental pollution could potentially cause mitochondrial abnormalities, metabolic disorders, cell apoptosis, etc., leading to DOR. Influenced by social factors and personal choices, the average childbearing age for women has increased. However, compared to other organs, the ovary ages significantly faster. This can not only trigger perimenopausal syndrome and ovarian-related diseases but also lead to a series of endocrine disorders such as Alzheimer’s disease, cardiovascular diseases, and osteoporosis (5). DOR has become one of the major challenges faced by clinicians today (6).

In clinical practice, many tests can be used to diagnose DOR, known as ovarian reserve tests (ORT). Among these, three are considered the most commonly used and effective: serum anti-Müllerian hormone (AMH), follicle-stimulating hormone (FSH), and antral follicle count (AFC). It is generally accepted that AMH levels <1.1 ng/ml, FSH ≥10 IU/L, and AFC <5–7 are considered indications for DOR. By investigating the effects of ROS and miRNA on AMH, FSH, and AFC, certain hormones can indirectly reflect the primordial follicle pool, thereby indirectly confirming the impact of ROS on female DOR.

Current treatment strategies for DOR primarily focus on symptom management, such as hormone therapy (HRT), dehydroepiandrosterone (DHEA), etc., as well as the timely provision of *in vitro* fertilization-embryo transfer (IVF-ET) for DOR patients with fertility desires. HT can alleviate menopausal symptoms and protect bone and cardiovascular health. DHEA exerts its androgenic function by being converted into testosterone and dihydrotestosterone in the ovary, and DHEA supplementation may improve oocyte quality in patients. IVF-ET, as a primary assisted reproductive technology, is one of the first-line treatments for infertile couples. However, long-term HRT may increase the risk of cardiovascular disease, breast cancer, etc. (7) Some clinical studies indicate that DHEA does not improve clinical pregnancy rates or live birth rates in DOR patients (8). The clinical pregnancy rate for IVF-ET is only 30-40% (9), and IVF cycles for some DOR patients end in poor ovarian response. Furthermore, older mothers who have undergone treatment face increased risks of placental abnormalities and dysfunction, preterm birth, neonatal asphyxia, etc. (10).

## 2 ROS

ROS do not refer to a specific substance but are a general term for many substances, primarily produced by mitochondria (11). The NOX family of NADPH oxidases and steroidogenic cytochrome P450 enzymes in ovarian tissue are also considered sources of ROS (12). ROS include the superoxide anion (O_2_•^-^), hydrogen peroxide (H_2_O_2_), and hydroxyl radical (•OH) (13), among others. O_2_•^-^ is harmful to the human body and can rapidly react with nitric oxide (NO) produced by endothelial cells, macrophages, etc., to form peroxynitrite anion (ONOO^-^), a highly cytotoxic strong oxidant (14). SOD catalyzes the dismutation of O_2_•^-^, thereby mitigating oxidative cellular damage.

The intracellular antioxidant system can eliminate free radicals and neutralize oxidants. The antioxidant system includes enzymes such as SOD, catalase (CAT), and glutathione peroxidase (GPx). In response to OS induced by free radicals, SOD appears to react rapidly by catalyzing the production of oxygen and H_2_O_2_ from peroxides. CAT catalyzes the decomposition of hydrogen peroxide into water and oxygen (15). GPx can reduce peroxides to less toxic compounds. It utilizes glutathione as a reducing agent to catalyze the conversion of H_2_O_2_ into water or corresponding alcohols. Therefore, this review synthesizes these fragmented insights to present a comprehensive and integrated narrative, aiming to elucidate the concept and fundamental functions of ROS, with a focus on the changes and roles of ROS physiology and pathology in DOR patients. This study facilitates readers’ understanding of the mechanistic relevance of ROS and how ROS can be utilized to treat DOR. The innovation of this work lies in its unified perspective, which bridges fundamental mechanisms with clinical significance, thereby guiding future diagnostic and therapeutic strategies in reproductive medicine.

## 3 ROS and ovarian physiology and follicular development

### 3.1 Physiological role of ROS in follicles

ROS has long been recognized primarily for its damage to cellular structures. However, increasing evidence suggests that physiologically active ROS are essential for normal follicular development, oocyte maturation, and ovulation. ROS is generated during normal physiological activities, and the body’s antioxidant mechanisms can maintain low concentrations of ROS, which is beneficial for cellular development. Physiological ROS is involved in the development of normal follicles, ovulation, and luteinization. Ovulation is stimulated by a pre-ovulatory surge of pituitary luteinizing hormone (LH). Genes associated with LH-induced ovulation include prostaglandin synthase 2 (Ptgs2), hyaluronic acid synthase 2 (Has2), tumor necrosis stimulus gene 6 (Tnfaip6), and CCAAT/enhancer binding protein β (Cebpb). These five genes should be significantly upregulated after LH stimulation in the absence of antioxidants, but their upregulation is markedly reduced following clearance of physiological ROS. H_2_O_2_ effectively induces pre-ovulatory follicular cloud expansion and is believed to upregulate the expression of ovulation-related genes. H_2_O_2_ is thought to mimic the ovulation-inducing behavior of LH. Cebpb, Has2, and Tnfaip6 exhibited levels comparable to LH under H_2_O_2_ induction, whereas Pgr and Ptgs2 showed lower induction levels compared to LH (16).

Moderate levels of ROS increase due to growth factors, ischemia, and wound injury, promoting angiogenesis. The vascular system plays a key role in ovarian development and normal ovarian function. The vascular system provides the necessary supply of gonadotropins, growth factors, oxygen, lipids, and steroid precursors for folliculogenesis and oocyte maturation and removes waste products generated by active follicular growth and metabolism (17). Regulating angiogenesis to improve follicular development can thereby restore ovarian reserve function in DOR patients. In endothelial cells and smooth muscle cells, ROS is influenced by various vascular stimuli, including angiopoietin-1 receptor, platelet-derived growth factor (PDGF), and vascular endothelial growth factor (VEGF). The four families of NADPH oxidase (NOX) are the main sources of ROS in the vasculature: NOX1, NOX2, NOX4, and NOX5 (18). Exogenous ROS can increase VEGF or VEGFR2 expression. There is a positive feedback mechanism between ROS and VEGF (19); VEGF can induce angiogenesis (20). ROS produced by NOX4 regulates hypoxia-inducible factor 1 alpha (HIF1α). In endothelial cells, HIF1α induces VEGF expression and can reduce mitochondrial respiratory capacity. Therefore, ROS produced by NOX4 at normal levels can dynamically regulate endothelial cell stability, thereby promoting angiogenesis (21).

ROS mainly coordinates cell function by regulating different signaling pathways, acting as a messenger between the inside and outside of cells. The ROS-mediated PI3K/AKT, FOXO, and p53 signaling pathways will be elaborated in the following sections.

### 3.2 Pathological role of ROS in follicles

#### 3.2.1 Granulosa cells

Granulosa cells (GCs), as an important component of the follicular microenvironment, are required to take up glucose due to the poor glucose absorption capacity of oocytes (22). Jiaqi Wu et al. used H_2_O_2_ combined with GCs to simulate ovarian OS status. The results showed a decrease in Glycerol-3-phosphatidyltransferase 1-like (GPD1L) content in GCs. Pearson correlation coefficient tests showed a positive correlation between GPD1L and AMH (23). This confirms that AMH is influenced by GCs, and ROS can damage GCs, thereby affecting AMH levels; decreased AMH levels reduce ovarian ovulation. Overexpression of MYBL2 promotes GCs’ proliferation.

#### 3.2.2 Mitochondrial damage

Mitochondria, one of the most abundant organelles in the human body, are referred to as the “powerhouses” due to their critical role in cellular metabolism and energy production. Mitochondria possess a unique dual-mode structure, with their inner membrane forming cristae that increase surface area. They participate in respiration, ATP synthesis, cellular redox reactions, Ca2+ homeostasis, Fe-S cluster biosynthesis, steroid synthesis, and apoptosis induction. Mitochondria generate ROS by consuming oxygen through the mitochondrial respiratory chain. During human aging, ATP production decreases, ROS generation increases, and antioxidant defense capacity declines accordingly. Mitochondrial DNA (mtDNA) exhibits impaired integrity due to the lack of histone. The protein is protected and overlaps with the ROS generation site of the mitochondrial inner membrane, making it susceptible to ROS attack. Additionally, it lacks a DNA damage repair system and is close to the electron transport chain system that generates anaerobic free radicals (24). In aging, chronic inflammatory stimulation, and obesity, ROS increase, making mitochondrial DNA more susceptible to damage (25). Excessively high ROS levels induce increased ROS release in adjacent mitochondrial systems, forming a positive feedback loop. The elevated ROS can damage mitochondria, leading to organelle excitation of mitochondrial electrical signals, which further promotes ROS transmission (20). Mitochondrial damage affects cellular redox balance, and elevated mitochondrial ROS levels can trigger programmed cell death pathways, such as apoptosis or autophagy (26). Mitochondrial dysfunction is considered to be one of the main mechanisms leading to oocyte senescence and functional decline.

#### 3.2.3 Telomere

Telomeres are DNA-protein structures at the ends of chromosomes, composed of short tandem repeats of DNA and a multicomponent protein complex called shelterin, which are crucial for maintaining genomic stability. Telomeres shorten with cell division and thus are considered closely associated with ovarian aging. They are also damaged by OS and other endogenous events (27). Senescent cells damage telomeres, and the expression levels of telomerase, telomerase reverse transcriptase, and telomere repeat binding factors 1 and 2 (TRF1 and TRF2) are reduced (28).

G-quadruplex (G4), a four-stranded nucleic acid structure formed by guanine-rich DNA and RNA sequences through Hoogsteen hydrogen bonding, plays a crucial role in telomere elongation. However, guanine exhibits low redox activity and is highly susceptible to oxidation. Additionally, the high guanine content in telomeres makes them prone to oxidative damage induced by ROS (27). OS may also impair telomere-protecting proteins, such as the inhibitory effects of OS on TRF1 and TRF2, leading to their dissociation (29). The primary functions of TRF1 and TRF2 are to maintain the structural integrity and length of telomeres in eukaryotic cells. Telomere damage leads to persistent DNA damage and aging.

## 4 Mechanism by which ROS induces DOR

### 4.1 Physiological mechanism of ROS-induced DOR

#### 4.1.1 Aging

The female reproductive system, as the earliest part of the human body to undergo aging, begins to decline in oocytes as women enter their thirties—a phenomenon known as maternal aging. With advancing age, the local microenvironment of the ovaries changes, leading to a decline in both the quantity and quality of oocytes, ultimately culminating in menopause. Fibrosis is a hallmark feature of aging tissues, resulting from the replacement of parenchymal tissue by excessive extracellular matrix, primarily produced by activated fibroblasts and myofibroblasts. Zhang J et al. found that in aged cells, differentially expressed genes exhibit an up-regulation trend, with these genes primarily associated with cellular fibrosis. This further corroborates the hypothesis of increased senescent ovarian fibrosis by involving ROS pathways, TGF-β signaling pathways, and HIF-1 signaling pathways (30). Fibroblasts are a major source of ROS within the ovarian stroma. During ovarian aging, the accumulation of senescent fibroblasts leads to increased ROS production. This excessive ROS burden activates pro-fibrotic signaling pathways (e.g., TGF-β/Smad) and impairs the local antioxidant defense system, creating a feed-forward loop. The resulting disruption of extracellular matrix homeostasis promotes fibrosis, which further exacerbates tissue dysfunction and accelerates the decline of ovarian reserve (31). Age-related mitochondrial damage is also considered a primary cause of oocyte decline, as mitochondrial dysfunction leads to DNA damage and increased ROS production.

### 4.2 Acquired/environmental mechanisms of ROS-induced DOR

#### 4.2.1 Obesity

Obesity is currently diagnosed by measuring Body Mass Index (BMI), calculated as weight (kg) divided by height (m) squared. A BMI above 30 is commonly used to define obesity at the population level. Elevated serum levels of oxidized low-density lipoprotein (Ox-LDL) (32), advanced oxidation protein products (AOPP), and malondialdehyde (MDA) were detected in obese patients, while the levels of enzymes such as SOD, CAT, and GSH—which protect cells against OS—were reduced (33). Despite clear evidence of elevated MDA levels, some meta-analyses demonstrated no significant association between MDA levels and obesity-related outcomes. significant differences in systemic NO or GPx levels, highlighting the complexity of redox balance. This study suggests that in DOR pathology, local tissue OS may be more relevant than systemic circulation levels. Obesity can impair reproductive system development, primarily attributed to functional alterations in the hypothalamic-pituitary-ovarian (HPO) axis. In obese women, excessive lipid accumulation and elevated circulating leptin levels lead to higher insulin levels compared to normal-weight women. The increased insulin promotes androgen production in the ovaries, which are then rapidly aromatized into estrogen at the periphery, resulting in negative feedback of the HPO axis and inhibition of gonadotropin production (34). In obese women, leptin levels exceed the normal range, and it is currently widely accepted that excessive leptin levels can impair follicular development. Elevated levels of inflammatory markers and free fatty acids are associated with abnormal cumulus-oocyte complexes (35). High concentrations of pro-inflammatory factors can damage oocytes, and inflammatory markers are elevated in obese women (36), for example, TNF-α, IL-6, E-selectin, VCAM-1, and ICAM-1. Free fatty acid levels are susceptible to OS, which may generate ROS that can disrupt mitochondrial and endoplasmic reticulum function, leading to extracellular matrix electron accumulation and leakage, ultimately resulting in programmed cell death in various cells, including oocytes (37). Obesity is often associated with the pathogenesis of insulin resistance. Most obese patients exhibit hyperglycemia, and intracellular hyperglycemia leads to excessive production of ROS (38). Excessive ROS can induce insulin resistance and serine/threonine phosphorylation. This shift from tyrosine to serine/threonine phosphorylation of insulin receptor subunits (IRS) represents a key molecular switch linking OS to insulin response. Resistance, which in turn perpetuates a vicious cycle of metabolic dysfunction and inflammation in DOR patients.

An increasing body of evidence suggests that gut microbiota serves as a critical mediator linking oxidative stress with clinical features of DOR. Obesity is characterized by gut microbiota dysbiosis, which enhances intestinal permeability—a phenomenon commonly referred to as “leaky gut”—promoting the systemic circulation of bacterial lipopolysaccharide (LPS). LPS binds to LPS receptors, forming complexes that interact with Toll-like receptor 4 (TLR4) in macrophages and adipose tissue. This triggers a broad spectrum of TLR4-mediated signaling pathways, inducing inflammation. Additionally, the IRS-1/PI3K pathway is impaired, leading to compromised insulin signaling and exacerbating insulin resistance (39). Simultaneously, the reduction of short-chain fatty acids (SCFAs) (primarily acetic acid, propionic acid, and butyric acid) distributed in the cecum and colon can increase human energy expenditure through acetate and butyrate metabolism. Additionally, SCFAs bind to GPR41/43 receptors, where GPR41 may play a role in human satiety perception, while GPR43 is crucial in mediating acetic acid-induced anti-inflammatory stimulation, leading to upregulated FOXO1 expression and enhanced ovarian androgen synthesis (40). Furthermore, dysbiosis-induced alterations in bile acid metabolism and estrogen receptors (via β-glucuronidase) disrupt FXR/TGR5 signaling and the HPO axis. Inducing a hormonal imbalance is a hallmark of this disorder. This complex interaction positions the gut microbiota as a potential therapeutic target.

Obese individuals may exhibit hyperandrogenemia and insulin resistance. Androgens are essential components of the normal female reproductive system and serve as critical precursors for estrogen synthesis (41). Current evidence suggests that hyperandrogenemia may promote ROS formation (42). Whether hyperandrogenemia constitutes a cause or consequence of the pro-inflammatory state in the DOR remains a debated issue, likely involving a bidirectional relationship: androgens can amplify inflammatory responses, while accumulating evidence—such as inflammatory signaling enhancing steroidogenic enzyme activity in luteal cells—indicates that inflammation may directly drive androgen production. This self-sustaining cycle is highly likely to constitute the pathophysiological basis of this syndrome. However, hyperandrogenemia is closely associated with polycystic ovary syndrome (PCOS), while studies related to DOR are limited.

Obesity is believed to affect follicular cells, but it cannot serve as the sole criterion for diagnosing DOR in women. It must be recognized that although the mechanisms discussed above are biologically plausible, most clinical evidence remains correlational and derived from small sample sizes or cross-sectional studies. Large-scale randomized controlled trials are urgently needed to validate these findings and establish causal relationships.

#### 4.2.2 Cigarette

Cigarette smoke contains nicotine, an addictive substance that poses significant harm to human health. While most people are only aware of the lung and cardiovascular-related risks associated with smoking, few women recognize the negative impact of smoking on fertility. Clinical data demonstrate that women who actively smoke or are exposed to secondhand smoke have prolonged conception time, significantly reduced conception rates, and increased infertility risks compared to non-smokers without passive smoking exposure (43). Cigarette smoke contains numerous harmful chemicals and pro-oxidants that deplete antioxidants and promote ROS production. Nicotine induces oocyte apoptosis by inhibiting GCs and estrogen synthesis. Chen T et al. found that vaping activates the Hippo signaling pathway, triggering phosphorylation reactions. LATS2 and YAP, as components of the Hippo signaling pathway, with LATS2 located upstream of YAP, enable YAP phosphorylation. The reduction of non-phosphorylated YAP inhibits GCs’ growth and estrogen synthesis (44). Nicotine disrupts normal insulin signaling pathways and is positively correlated with central obesity (45).

#### 4.2.3 Chemotherapy

GCs are susceptible to chemotherapeutic agents, which may induce DNA double-strand breaks, and DNA damage can trigger apoptosis. Chemotherapeutic agents such as cyclophosphamide and cisplatin have been shown to significantly downregulate the expression of AMH, follicle-stimulating hormone receptor (FSHR), and estrogen receptor (ER) in mice, strongly suggesting ovarian reserve insufficiency. Following cisplatin treatment, there is a marked increase in lipid ROS accumulation in ovarian GCs. GPx4, a key enzyme for reducing intracellular lipid ROS, is downregulated in the ovaries by cisplatin (46). Cyclophosphamide can be converted into phosphamidic mustard and acrylamide through the intermediate 4-hydroxycyclophosphamide, inducing DNA cross-linking, blocking DNA replication, and exhibiting cytotoxic effects. The olefin derived from cyclophosphamide metabolism can increase ROS production through NADPH activation, leading to the development of DOR in females (47).

## 5 Signal pathways and molecular mechanisms

### 5.1 Abnormalities in ROS-mediated pathways

Although ROS plays important roles in the body, excessive ROS can induce ovarian diseases. Increasing age or external adverse factors can cause excessive ROS production in the body, leading to DNA oxidation, damage, apoptosis, etc.

**Figure 1:**
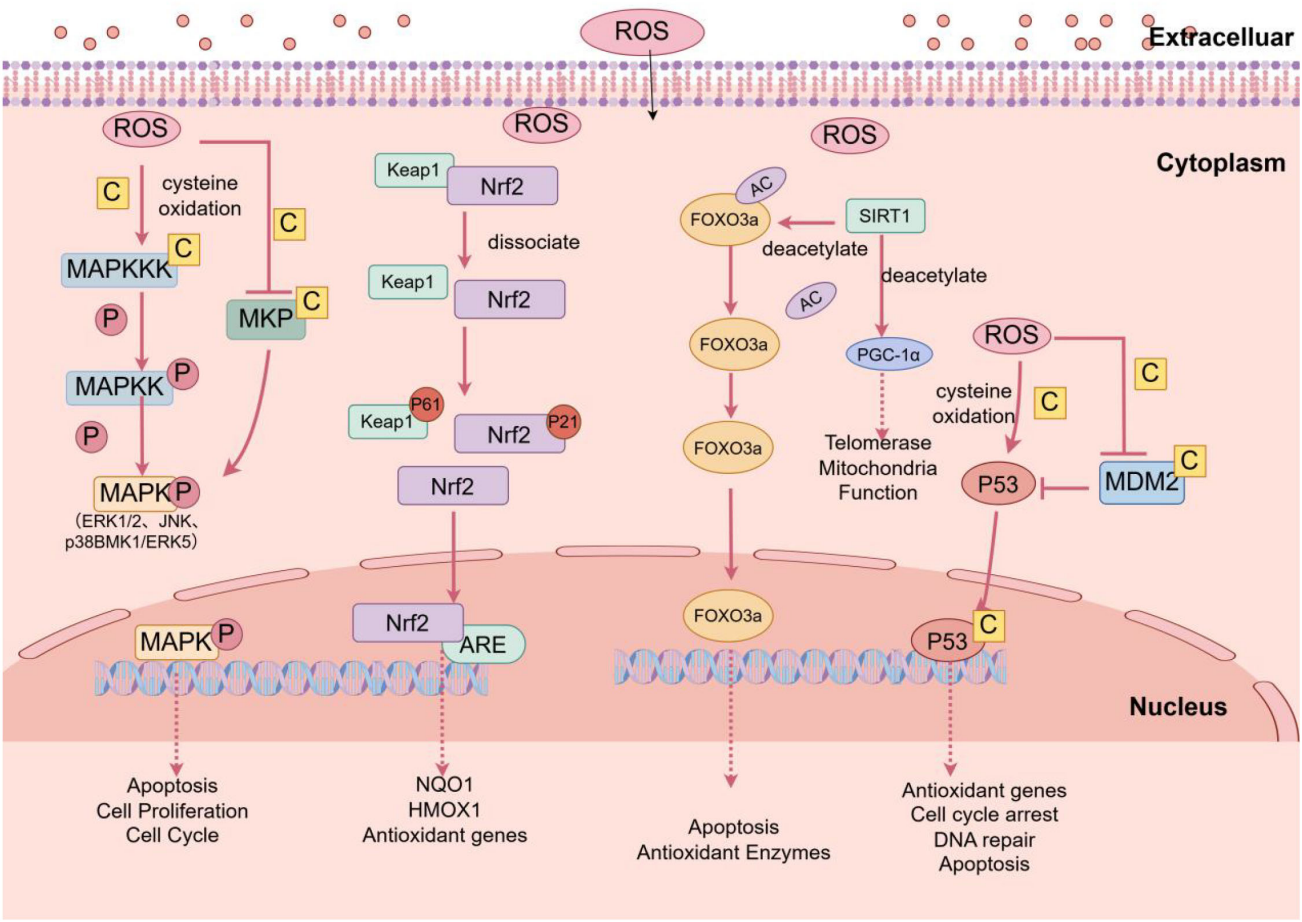
The role of signal transduction under excessive ROS on the ovary. The Keap1-Nrf2 structure is disrupted, resulting in partial p61-Keap1 and p21-Nrf2. Nrf2 enters the nucleus and binds to ARE. SIRT1 can deacetylate FOXO3a; FOXO3a induces antioxidant effects via SOD and CAT and regulates telomerase activity and mitochondrial function by modulating PGC-1α. ROS activates p53 and prevents MDM2 from degrading p53, promoting DNA repair and antioxidation. MAPK is activated by ROS, transmitting extracellular signals into the nucleus, promoting apoptosis. Image source: figdraw.

The preovulatory LH surge increases the levels of pro-inflammatory precursors in the ovary, leading to increased ROS content. The regulation of transcription factor nuclear factor erythroid 2-related factor 2 (Nrf2), SIRT1, mitogen-activated protein kinase (MAPK), p53, and other OS signaling pathways plays important roles in ovarian OS damage (48). Under basal conditions, most Nrf2 is bound to Kelch-like ECH-associated protein 1 (Keap1), while the remaining Nrf2 exerts antioxidant effects. Under OS, Nrf2 degradation decreases, accumulation increases, and nuclear translocation occurs. Nrf2 binds to antioxidant response elements (ARE) such as NADPH quinone oxidoreductase 1 (NQO1), heme oxygenase-1 (HMOX1), and ferritin heavy polypeptide 1 (FTH1) (49), conferring cytoprotective effects, with NQO1 and HMOX1 having antioxidant enzyme functions. OS causes redistribution of SIRT1 (50). SIRT1 enhances antioxidant effects by increasing SOD, CAT, and GPx1 and also deacetylates and activates endothelial nitric oxide synthase (eNOS), increasing NO content (51, 52). ROS activates p53 by oxidizing specific cysteine residues. p53, as the “guardian of the genome,” limits DNA damage after OS (53). Activated p53 translocates to the nucleus to induce cell cycle arrest to allow DNA repair or induce apoptosis if DNA cannot be repaired. ROS can also oxidize specific cysteine residues in MDM2, reducing MDM2-mediated degradation of p53, leading to increased p53 levels (54). ROS directly activates MAP kinase kinases (MAPKKKs), leading to MAP kinase kinases (MAPKKs), and ultimately forming MAPKs. ROS can also indirectly inhibit MAPK phosphatases (MKPs), causing sustained activation of MAPKs. MAPKs include ERK1/2 (extracellular signal-regulated kinases), c-Jun N-terminal kinase (JNK), p38 (p38 kinase), and BMK1/ERK5 (big MAP kinase 1). p38 and ERK1/2 can, in turn, induce ROS production. Among these, excessive ROS activates the ASK1-JNK pathway. The main mechanism is that apoptosis signal-regulating kinase 1 (ASK1), a serine/threonine protein kinase involved in cell differentiation and apoptosis, dissociates from Trx-1 under conditions of excessive ROS and induces apoptosis by activating JNK in the MAPK pathway, reducing the number of oocytes in the ovary (55) (**Figure 1**).

**Figure 2:**
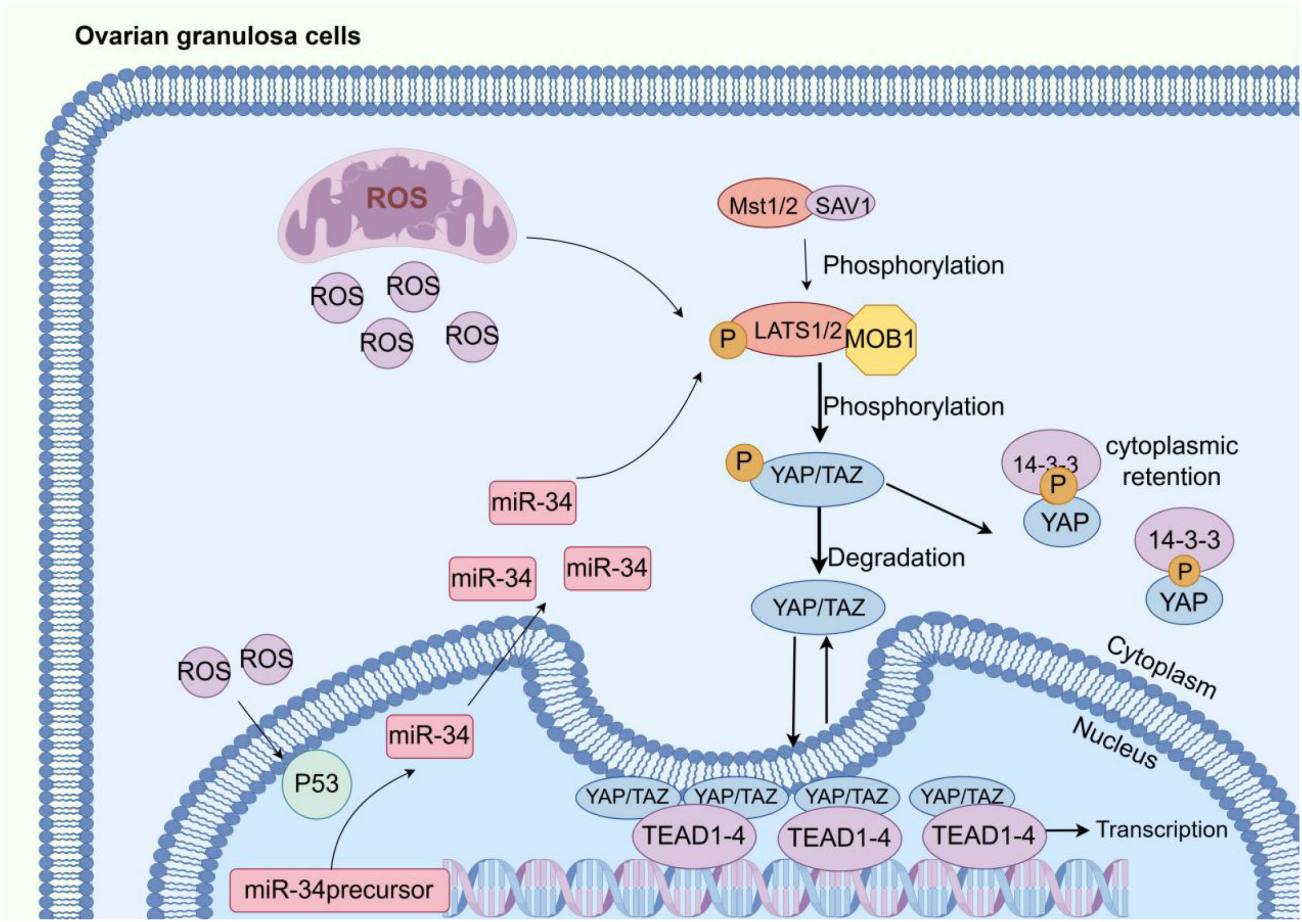
ROS–miRNA–Hippo–GC apoptosis axis visual model. When the Hippo pathway is activated, the MST1/2-SAV1 complex phosphorylates and activates the LATS1/2-MOB1 complex. The activated LATS1/2-MOB1 complex phosphorylates YAP and TAZ, leading to their cytoplasmic retention by 14-3–3 proteins or degradation. When the Hippo pathway is inactive, unphosphorylated YAP and TAZ translocate into the nucleus and bind to TEAD1–4 to form a complex. Reactive ROS generated in mitochondria activate p53, which upregulates miR-34. Dysregulated miRNAs synergize with ROS to activate the Hippo pathway. Image source: figdraw.

In addition, the Hippo pathway is closely linked to ROS and is considered to be activated by ROS. However, the specific mechanism has not been clearly explained yet. Current research suggests that ROS is related to the activation of LATS1. To meet the pregnancy needs of DOR patients, ovarian stimulation and *in vitro* fertilization (IVF) are often used as diagnostic and therapeutic approaches (56). Precisely because of DOR, IVF is not always successful for DOR patients. Some research has focused on the Hippo pathway, aiming to inhibit it to prevent it from suppressing organ growth, thereby promoting follicular development. The Hippo pathway, also known as the Salvador/Warts pathway, was first discovered in *Drosophila melanogaster* as a highly conserved pathway regulating organ size control. It plays important roles in follicular proliferation and steroidogenesis within the ovary. The core components of the Hippo pathway include mammalian STE20-like kinase 1/2 (MST1/2), protein Salvador homologue 1 (SAV1), MOBKL1A/B (MOB1A/B), large tumor suppressor kinase 1/2 (LATS1/2), Yes-associated protein 1 (YAP), WW-domain-containing transcription regulator 1 (TAZ), and the transcriptional enhancer-associated domain (TEAD) family (57). YAP and TAZ bind to TEAD to regulate processes such as cell proliferation and apoptosis (58) (**Figure 2**).

Once ROS becomes uncontrolled and levels rise within cells, the balance between oxidation and antioxidation in the body is lost. Excessive accumulation of H_2_O_2_ leads to OS. MST1/2 in the Hippo pathway is activated under OS stimulation and phosphorylates FOXO3 (59), leading to apoptosis (59), as shown in **Table 1**. In the ovary, Lv X et al. found that this behavior led to an increase in the number of primordial follicles and a decrease in the number of primary follicles by knocking down YAP1, while the results of YAP1 overexpression were opposite (60). Studies have proven that YAP1 primarily induces the development of primordial follicles into primary follicles. Specific loss of YAP in primordial follicles leads to apoptosis, thereby affecting ovarian reserve (61). Previously used but now less common treatments like ovarian wedge resection, diathermy, and laser ovarian drilling are also suspected to achieve their therapeutic effects by disrupting the Hippo pathway (62).

**Table 1:** Association between hippo signaling pathway and ROS OS.

author	mechanism	bibliography
Liying Luo et al.	Activation of the Hippo signaling pathway in conjunction with ferroptosis may enhance ROS production and lead to more cellular damage.	(63)
Tao Zhang et.al	Extracellular matrix (ECM) stiffness is involved in the regulation of extracellular ROS levels by modulating YAP activity.	(64)
Jingzeng Cai et al.	The Hippo-YAP/TAZ pathway has been shown to prevent OS O_2_ levels by metabolizing through a series of operations, such as the breakdown of glucose to regulate H.	(65)
Zhenzhen Zhang et.al	YAP is located downstream of NOX4-derived ROS. NOX4 can activate the YAP pathway via ROS.	(66)
Hsin-Yi Huang et al.	YAP promotes ROS accumulation by down-regulating ROS scavenging genes such as GPx2. YAP disrupts intracellular ROS homeostasis via the DNp63-GPx2 axis.	(67)
Yu HF et.al	Yap inactivation leads to a massive accumulation of intracellular ROS through the defective Rrm2/GR/GSH pathway.	(68)
Hiroki Nagai et al.	Autophagy defects cause ROS-dependent hyperregeneration, and the autophagy substrate Ref(2)P/p62 accumulates during autophagy defects, thereby inactivating Hippo signaling.	(69)
Bae SJ et.al	Spatholobi Caulis inhibits ROS generation and mitochondrial dysfunction *in vitro* by activating the LKB1-AMPK pathway and the Hippo-Yap pathway.	(70)
Wehling L, et al.	Acetaminophen administration leads to YAP inactivation (early) and YAP activation (late) through ROS axis induction.	(71)
Xiang J et al.	Activated TAZ promotes the expression of epithelial membrane protein 1 (EMP1), which then upregulates the level of NADPH oxidase 4 (NOX4), which increases the level of lipid ROS in cells and induces iron death.	(72)
Tao Hu et al.	Decreased expression leads to increased intracellular ROS levels, which further activate the Hippo-Yap/TAZ signaling pathway, and Yap/TAZ expression is decreased when the pathway is activated.	(73)
Hui Yu et al.	Cellular ROS levels downregulate YAP mRNA expression. GP4, an important antioxidant enzyme, is dependent on YAP activation for its expression, and thus, YAP is counterproductive to oxidation.	(74)
Maohua Wang et al.	YAP overexpression significantly decreased ROS accumulation, and the downregulation of YAP expression increased ROS accumulation.	(75)
Yongyun Li et al.	Accumulation of ROS leads to senescent cell dysfunction by triggering NF-κB signaling.	(76)
Xiao Han et al.	Circulating ROS mediates cardiac insufficiency after bilateral renal ischemia-reperfusion injury via activation of cardiac Hippo pathways.	(77)
Jixin Dong et al.	YAP S127 phosphorylation mediates the growth inhibitory output of the mammalian Hippo pathway, which is suspected to be involved in the control of organ size.	(78)

### 5.2 The interaction between inflammatory factors and ROS

Chronic inflammation accelerates the aging process of the body. With advancing reproductive age, the secretion of pro-inflammatory cytokines such as IL-6, IL-1β, and TNF-α in the ovaries significantly increases. These cytokines, acting as pro-inflammatory mediators, play a crucial role in ovarian inflammation by inducing ROS production through NADPH oxidase. ROS can amplify the effects of inflammatory factors (79); excessively high ROS levels enhance the OS environment in the ovaries and trigger protein phosphorylation in the NF-κB signaling cascade. The activated NF-κB signaling pathway promotes the release of inflammatory cytokines (80). ROS can induce inflammation, affect the activity of mitochondrial respiratory chain complexes, lead to oxidative phosphorylation (OXPHOS) issues, and reduce ATP production (46). When treating patients with DOR, our goal is not to eliminate ROS but to restore ROS to a normal physiological level. However, there is insufficient research on the dosage of drugs required to restore ROS to an ideal level.

## 6 Clinical studies of antioxidants

**Figure 3:**
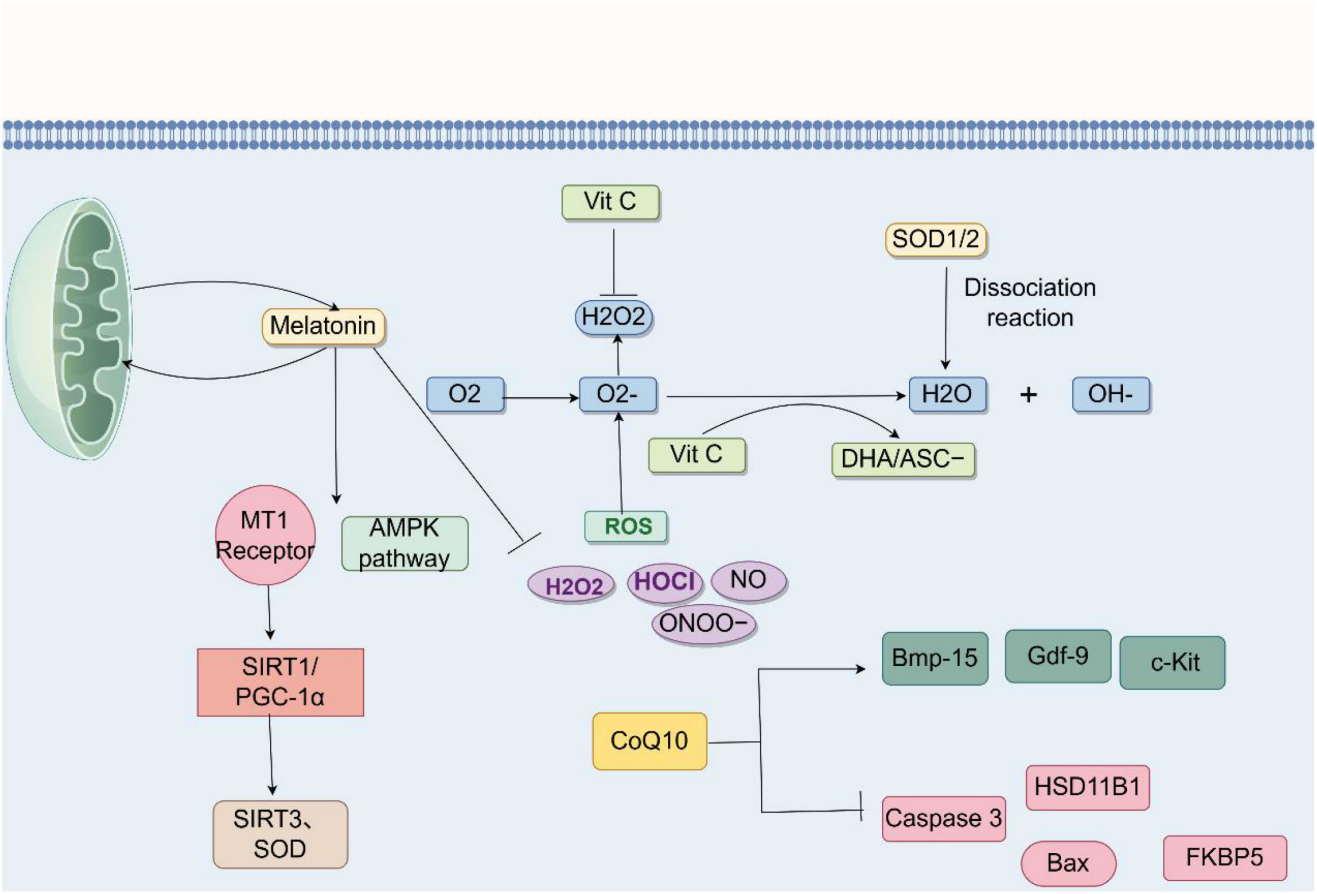
Therapeutic roadmap (Vitamin C/melatonin/CoQ10 modulation). Image source: figdraw.

Excessive ROS in the body causes OS, generating free radicals that damage GCs and severely affect ovarian development. Scavenging free radicals and using antioxidants are widely considered important factors in delaying ovarian aging and preserving reserve function. When ROS is upregulated beyond the normal range, it dysregulates miRNAs. Therefore, antioxidants can be used to restore excessive ROS and dysregulated miRNAs to normal ranges, avoiding the decline in ovarian reserve function that causes DOR (**Figure 3**).

### 6.1 Vitamin C (ascorbate)

Vitamin C is a water-soluble compound that can catalyze enzymatic reactions for certain hormone activities. Due to its biological antioxidant properties, it is used as an antioxidant to reduce oxidative damage. Normal concentrations of vitamin C have been proven to reduce intracellular ROS levels, inhibit lipid oxidation by reducing biophenol radicals, and help maintain intracellular redox balance (81). However, high concentrations of vitamin C can enhance ROS production. pSTAT3, as a downstream signal of ROS, is also inhibited by vitamin C (82). Anatoly Zhitkovich believes that vitamin C is more efficient at scavenging the oxygen-free radical O_2_•^-^ compared to the similarly acting glutathione. O_2_•^-^, as a primary ROS, reacts with NO to produce ONOO^-^. Cellular enzymes SOD1/2 produce H_2_O_2_ through dismutation (83). Vitamin C also helps the body recycle vitamin E and glutathione (84). Vitamin C easily auto-oxidizes *in vitro* to O_2_•^-^ and ascorbate anion. Vitamin C can decompose into an electron and ascorbate anion; the electron combines with O_2_•^-^ to scavenge free radicals, or it can lose a second electron to form its oxidized form, dehydroascorbic acid (DHA) (85). DHA has vitamin C activity when administered orally (86). However, current research on vitamin C and ROS is not extensive and is controversial.

### 6.2 Melatonin

Melatonin (5-methoxy-N-acetyltryptamine) is a natural endogenous hormone secreted by the pineal gland. As a powerful antioxidant, it possesses stronger antioxidant capacity than traditional antioxidants like vitamins C and E, glutathione, etc. It easily scavenges most toxic free radicals, such as neutralizing O_2_•^-^, •OH, singlet oxygen (¹O_2_), H_2_O_2_, hypochlorous acid (HOCl), NO, and ONOO^-^ (87); and it stimulates antioxidant enzyme activity like GPx and SOD to achieve antioxidant effects. Interestingly, melatonin improves mitochondrial function by protecting it, and improved mitochondria can, in turn, promote the synthesis of melatonin synthase (88). Melatonin has also been confirmed to be present in the follicular fluid within GCs (89). Melatonin levels in human follicular fluid are highly correlated with ovarian reserve. Furthermore, melatonin declines with age, and many people experience low oocyte quality leading to DOR due to decreased melatonin. The positive effects of melatonin on oocytes can be mediated by the MT1 receptor and AMPK pathway. Melatonin can activate the SIRT1/PGC-1α pathway and upregulate SIRT3 and SOD (90). After knocking out the MT1 receptor, SIRT1 levels decrease. SIRT1, as a ROS transcription factor, enhances antioxidant effects by increasing SOD, CAT, and GPx1, leading to reduced ROS and improved oocyte quality. It is speculated that melatonin maintains ovarian function through antioxidant actions via the MT1 receptor and AMPK pathway (91).

### 6.3 Coenzyme Q10

CoQ10 is a fat-soluble, lipophilic molecule located in the mitochondrial inner membrane and involved in ATP production. It can improve the mRNA expression of FSHR and PCNA and directly target mitochondria to remove excess ROS, therefore being widely cited in the treatment of DOR. The decline in CoQ10 levels roughly coincides with the age of declining female fertility, suggesting CoQ10’s contribution to ovarian development (92). CoQ10 can not only act as an antioxidant but can also turn into a pro-oxidant at different concentrations. Lower CoQ10 reduces ATP production and increases OS (93). In Guangyao Lin’s experiment, it was proven that after supplementing with CoQ10, the expression of apoptosis-related genes Caspase 3, Bax, HSD11B1, and FKBP5 can be significantly reduced (94). Some studies have confirmed that coenzyme Q10 stimulates the expression of Bmp-15, Gdf-9, and c-Kit, which are representative regulatory factors for primordial follicle activation and follicle development, and KL/c-Kit is very important for follicle development (95).

### 6.4 Others

Lifestyle modifications offer promising approaches to managing OS in DOR, although their effects are complex. While exercise may lead to a mild increase in ROS levels, physical and mental practices such as yoga appear beneficial, with improved antioxidant systems (96). These changes seem to result from regular physical activity that reduces OS, thereby aiding in the restoration of normal endocrine function and fertility. Numerous natural plant extracts have been demonstrated to possess antioxidant properties, including various natural plant metabolites such as polyamines, resveratrol, ferulic acid, L-carnitine, and lotus urine. Resveratrol, in particular, inhibits ROS production and NLRP3 inflammation (97). Downstream activation of the nucleolar complex (98).

## 7 Challenges and future outlook

Although significant progress has been made in understanding the mechanisms of ROS in DOR, and intervention strategies based on this knowledge show broad promise, the field still faces multiple challenges. Clarifying these bottlenecks and defining future research directions are crucial for advancing the translation of basic research findings into clinical practice. The boundary between ROS’ physiological role as signaling molecules and its pathological role as damage factors remains blurred, and the differential sensitivity of ROS to different stages of follicular development has not been systematically elucidated. Future research should transcend the traditional HPO axis to incorporate a multisystem perspective. Investigating the complex interactions between the HPO axis and peripheral systems—such as the gut-liver-ovarian axis, thyroid and adrenal disorders, and metabolic disturbances (e.g., impaired glucose tolerance and type 2 diabetes)—will be crucial. Understanding how protein-energy balance and body weight influence these networks may reveal novel therapeutic targets for preserving ovarian reserve. Existing clinical studies are predominantly small-sample, single-center, observational studies, with a limited number of randomized controlled trials.

## 8 Conclusion

The role of ROS in DOR has evolved from the simplistic concept of ‘oxidative damage’ to a systemic pathological model encompassing signal network dysregulation, energy metabolism reprogramming, cell fate determination, and tissue microenvironment remodeling. Future research should focus on exploring and reconstructing the precise regulatory network of ovarian redox homeostasis. This will transform ROS from a passive damage repair mechanism to an active homeostatic maintenance system and shift from single-layer antioxidant supplementation to multi-level, individualized precision regulation. Understanding the roles of ROS in DOR provides valuable insights for developing new diagnostic and therapeutic strategies. Targeting these pathways could lead to more effective treatments for DOR and improve the reproductive outcomes of affected women.
